# Student Response to Remote-Online Case-Based Learning: A Qualitative Study

**DOI:** 10.2196/mededu.5025

**Published:** 2016-03-22

**Authors:** Peter Nicklen, Jennifer L Keating, Stephen Maloney

**Affiliations:** ^1^Monash UniversityDepartment of PhysiotherapyMelbourneAustralia

**Keywords:** case-based learning, Web conferencing, remote-online case-based learning, student satisfaction, perceived depth of learning

## Abstract

**Background:**

Case-based learning (CBL) typically involves face-to-face interaction in small collaborative groups with a focus on self-directed study. To our knowledge, no published studies report an evaluation of Web conferencing in CBL.

**Objective:**

The primary aim of this study was to explore student perceptions and attitudes in response to a remote-online case-based learning (RO-CBL) experience.

**Methods:**

This study took place over a 2-week period in 2013 at Monash University, Victoria, Australia. A third year cohort (n=73) of physiotherapy students was invited to participate. Students were required to participate in 2 training sessions, followed by RO-CBL across 2 sessions. The primary outcome of interest was the student feedback on the quality of the learning experience during RO-CBL participation. This was explored with a focus group and a survey.

**Results:**

Most students (68/73) completed the postintervention survey (nonparticipation rate 8%). RO-CBL was generally well received by participants, with 59% (40/68) of participates stating that they’d like RO-CBL to be used in the future and 78% (53/68) of participants believing they could meet the CBL’s learning objectives via RO-CBL. The 4 key themes relevant to student response to RO-CBL that emerged from the focus groups and open-ended questions on the postintervention survey were how RO-CBL compared to expectations, key benefits of RO-CBL including flexibility and time and cost savings, communication challenges in the online environment compared to face-to-face, and implications of moving to an online platform.

**Conclusions:**

Web conferencing may be a suitable medium for students to participate in CBL. Participants were satisfied with the learning activity and felt they could meet the CBL’s learning objectives. Further study should evaluate Web conferencing CBL across an entire semester in regard to student satisfaction, perceived depth of learning, and learning outcomes.

##  Introduction

Case-based learning (CBL) is an educational approach where students work in collaborative groups to solve a series of problems presented in a context that students are likely to encounter in practice [1]. CBL typically involves face-to-face interaction in small groups with a focus on self-directed study [2]. In CBL, students are responsible for identifying knowledge deficits relating to the case; this encourages learners to develop and manage their own learning goals and strategies needed for lifelong learning [3]. Problem-based learning (PBL) is a different instructional method in which students in collaborative groups learn through facilitated problem solving, working through a complex problem that does not have a single correct answer [3]. Like CBL, PBL has a focus on self-directed learning; however, a key difference suggested by Savery [4] is that CBL may influence the learner’s role in setting the goals and outcomes for a problem given its structured nature around a case presentation. Given the similarities of the two methods, we feel the findings of our study would apply equally to PBL.

Computer-assisted learning (CAL) is the implementation of computer technology to create a rich environment for active learning [5]. CAL has the potential to facilitate active, self-directed learning and enhance student knowledge and understanding [6] and may complement the current CBL process [1]. Lewis et al [6] reviewed 25 papers evaluating the use of CAL in nursing education and highlighted the overall poor quality of the studies and need for further investigation. In a review of 6 papers, Cook [1] concluded that Web-based PBL in nursing education encourages student autonomy and provides flexibility and opportunities for discussion. However, a paucity of relevant research was also noted [1]. Other benefits of Web-based learning (WBL) include the reduction in barriers of distance and time and the option to individualize learning opportunities for students [7]. Major challenges reported include technical difficulties and slow download speeds, costs associated with setting up and participating in WBL activities, and the potential for students to experience social isolation [1,7]. Cook [7] defined WBL in medical education as any educational interventions that make use of the Internet (or a local intranet) and broadly categorized these into tutorials, online discussion groups, and virtual patients. In this study, Web conferencing was used to integrate CBL and WBL, which we labeled remote-online CBL (RO-CBL). To our knowledge, no published studies report an evaluation of Web conferencing in CBL.

Following a systematic review, Crawford [1] concluded that CAL could be beneficial within the context of CBL. Crawford [1] recommended that carefully planned training sessions are required to reduce some of the challenges of implementing a Web-based activity and further research is required to determine the effectiveness, efficiency, and sustainability of Web-based CBL. Student satisfaction was not an outcome of interest in that review. The authors of 4 randomized controlled studies [8-11] concluded that Web-based CBL is comparable to face-to-face CBL in student learning outcomes. However, training in preparation for Web-based activities was either not reported [9,10] or not reported with the detail required for replication [8,11]. None of the 4 trialed interventions incorporated online discussions in the form of Web conferencing.

Valaitis et al [12] also noted a lack of research into Web conferencing in health science education. Yeung [13] compared Web-conferenced learning to face-to-face learning and concluded that both approaches produce comparable learning outcomes. Anecdotally, participants generally have a high level of satisfaction with Web-conferenced learning [12,14-16]. However, it should be emphasized that Web-conferenced learning is a generic term incorporating a wide range of online systems with varying levels of functionality ranging from simple synchronous communication tools to high-technology replication of clinical environments such as virtual patients [17]. A pilot study by our research team provided data showing that RO-CBL results in comparable learning outcomes when compared to face-to-face CBL. Students faced connection issues during the pilot study, which resulted in CBL taking longer to complete; this made communication difficult and resulted in low student satisfaction. Raupach [10] also reported low student satisfaction with an online collaborative teaching module that incorporated live chats, asynchronous group discussions, and document exchange. Although both our study and the study by Raupach [10] indicated that learning outcomes for Web-based and face-to-face learning activities were comparable, low student satisfaction was a common theme with the Web-based medium. Student satisfaction is as important as learning outcomes when evaluating the effectiveness of new approaches to teaching and learning.

In an attempt to combat low student satisfaction, we explored student needs and preconceptions of RO-CBL prior to a trial of the learning activity. One-third of the participants (23/71, 32%) were hesitant to move to an online format. Prior to training, students reported that they understood how RO-CBL worked; however, they were unsure how it would work in practice. This could account for the hesitation to move to the online format. Training sessions were then designed to target improved understanding of RO-CBL and the way it works in practice. Following the training sessions, there was a significant shift with participants reporting increased knowledge about RO-CBL: how it would work in practice, how they could meet the learning objectives using this new mode of learning, and how it might be used effectively in the future. Participants were also confident using the Web conferencing software. It was hypothesized that targeted training when introducing Web-based learning might reduce resistance to change, enhance the potential for student satisfaction, and improve the learning experience.

This study was designed to investigate student feedback following an RO-CBL trial. The study aimed to explore how RO-CBL compared to preconceptions, evaluate overall student satisfaction and perceived depth of learning, and identify possible barriers to the uptake of Web-based CBL by understanding the student experience.

##  Methods

### Design

This study used a mixed methods framework (focus groups and surveys) to assess the perceived value of RO-CBL after exposure to activities designed to build skills for participating in Internet-based CBL. Ethics approval was obtained through the Monash University Human Research Ethics Committee (Ethics CF 13/456-2013000200).

### Participants

**Figure 1:**
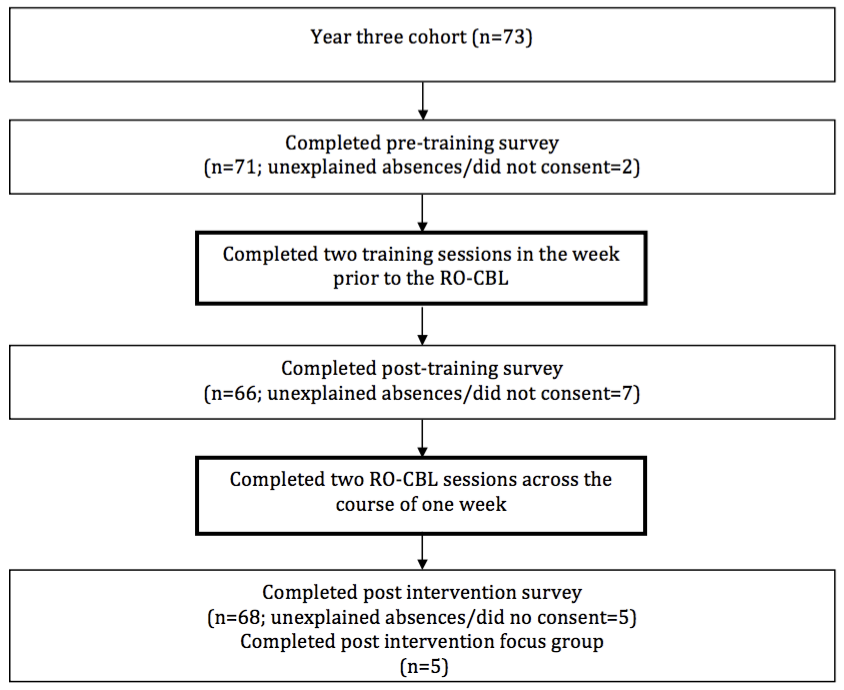
Participant flowchart and data collection points.

This study took place over a 2-week period in 2013 at Monash University, Victoria, Australia. All students are required to complete the RO-CBL as part of the third year curriculum, and the entire cohort (n=73) was invited to participate in the study. An independent research assistant recruited participants through face-to-face delivery and distribution of an information package with an explanatory statement. Students who did not consent to the study were not required to complete the outcome measures questionnaires that were administered. Figure 1 summarizes participant flow and data collection process.

### Intervention

In the Monash physiotherapy program, the two-part CBL is completed in small groups of 4 to 6 students on campus over the course of one academic week. Students are required to assign members of their CBL team the roles of leader, scribe, and recorder. The leader’s role is to keep the discussion on topic and make sure the CBL is completed within the designated time frame. In the traditional CBL format, the scribe makes notes of discussions on a whiteboard, and the recorder transcribes the whiteboard notes to a format that can be distributed to other students. In earlier years of the program, students learn to conduct effective CBL with dedicated facilitators. By the third year, students are reasonably proficient, and only one academic facilitator is required to monitor and provide guidance to all CBL groups, encouraging student-led discovery and achievement of learning objectives.

Part 1 of the case begins with a trigger (brief scene setting), followed by the case details: history, physical examination outcomes, further information, actions arising (eg, treatments, tests), reassessment, and closure, all of which are scripted to simulate a typical client interaction. Students are required to answer questions presented throughout the case and produce a problem list, which helps guide discussion. Students identify additional learning they are required to seek out in order to understand the case (learning issues); these questions and topics are then researched by individuals or groups of students during the week and presented to the group in Part 2.

During the first semester of 2013, third year students participated in an RO-CBL. Students were required to attend two training sessions the week prior to the RO-CBL, which then took place over the course of one week and required students to complete Part 1 and Part 2 online in groups of 4 to 6. Students were not required to be on campus, allowing them to participate from a location of their choice. Students supplied their own computers, microphones, and video cameras. One academic facilitator was responsible for monitoring all groups.

### Web Conferencing Software

The Web conferencing software (Google Hangouts) allowed students to interact via webcam and microphone as well as access and work collaboratively on the same document. This shared document saved automatically and could be viewed by students at any time during or after the CBL. Students were also able to upload documents and present findings to others in the group using the screen sharing function. All students used the same Web conferencing software.

### Training Procedure

Two training sessions occurred prior to the RO-CBL. The first was a 60-minute information session run by the RO-CBL facilitator. During this session, students were shown how to set up an RO-CBL and use the key functions with a step-by-step demonstration of the Web conferencing software. The second session was a 30-minute self-directed session. Students were required to set up an RO-CBL and complete a checklist demonstrating that they had mastered the key features of the Web conferencing software. Two assistants were available to answer questions and help with issues that arose. Students also had access to the sessions in the weeks prior to the RO-CBL and were encouraged to explore them during this time.

### Outcomes

The primary outcomes of interest were the student perceptions and attitudes in response to an RO-CBL learning experience, collected via focus group and survey following participation in the RO-CBL. Postparticipation assessment to understand the student experience explored student satisfaction, perceived depth of learning, and how RO-CBL compared to preconceptions to identify possible barriers to the uptake of Web-based CBL.

The survey was distributed on the day following Part 2 of the RO-CBL. It explored opinions about the experience of participation and the willingness to incorporate RO-CBL into the curriculum. Responses were provided using a 5-point Likert scale ranging from “strongly disagree” to “strongly agree.” There were 3 open-ended questions: What did you like about the RO-CBL experience? How could your RO-CBL and/or training be improved? How did the RO-CBL experience compare to your preconceived thoughts? Data contributing to an economic analysis of RO-CBL compared to traditional face-to-face CBL were also collected [18]. The survey was distributed and collected by a research assistant who was not involved in teaching or assessing participants. Students who completed both RO-CBL sessions were invited to participate in optional focus groups by a second independent research assistant who also ran the session. Participant selection was based on order of response to the invitation.

Two focus groups were conducted approximately 2 weeks after the intervention period and ran for approximately 30 minutes each. Inquiry was aligned with the survey and allowed for greater depth of discussion. Survey responses and focus group data were collected prior to analysis. Textbox 1 presents the focus group questions that served as prompts for discussion. An external transcription service was used to deidentify and transcribe recordings.

**Focus group questions.**

Questions guiding focus group discussion:

- What did you like about the online CBL experience?
- How does this experience compare to your preconceptions of online CBL?
- What could have improved the online CBL experience?
- How do you see online CBL being used in the future?

### Data Analysis

Thematic analysis was used to interpret the focus group transcripts and responses to the open-ended questions. Two independent researchers classified and grouped segments of text to create and define themes that emerged from the data [19]. Responses to both the focus group and open-ended questions were pooled prior to coding and development of themes. Once patterns were identified after coding, the researchers worked together to reach a consensus on the final themes. Responses to Likert scales were summarized using percentage and number of participants selecting each response option.

## Results

### Participants

All students enrolled in the third year of a Bachelor of Physiotherapy program at Monash University, Victoria, Australia, in 2013 were invited to participate. All 73 students were required to attend the two training sessions and complete the two parts of the RO-CBL in the designated times. Of these, 68 students completed the postintervention survey (nonparticipation rate 8%) and 5 participated in the focus groups.

### Survey Responses

Almost all participants responded to the questions “What did you like about the RO-CBL experience?” (67/68, 99%) and “How did the RO-CBL experience compare to your preconceived thoughts?” (66/68, 97%). Fewer participants (55/68, 81%) responded to the question “How could your RO-CBL and/or training be improved?” See Table 1 for responses to the postintervention surveys.

**Table 1:** Postlearning activity survey responses.

	Strongly disagree n (%)	Disagree n (%)	Neutral n (%)	Agree n (%)	Strongly agree n (%)
I met the CBL's learning objectives via RO-CBL.	0 (0)	1 (1)	14 (21)	30 (44)	23 (34)
I could envisage RO-CBL being used in the future.	1 (1)	2 (3)	15 (22)	24 (35)	26 (38)
I would not like RO-CBL to be used in the future.	17 (25)	23 (34)	18 (26)	6 (9)	4 (6)
I did not enjoy trialing RO-CBL.	28 (41)	22 (32)	10 (15)	7 (10)	1 (1)
With some more practice, I believe I would prefer RO-CBL to face-to-face CBL.	3 (4)	13 (19)	25 (37)	12 (18)	15 (22)
Google Hangouts was difficult to use.	23 (34)	29 (43)	12 (18)	3 (4)	1 (1)

The 4 key themes relevant to student response to RO-CBL that emerged from the focus groups and open-ended questions on the postintervention survey were expectations, benefits of RO-CBL, communication, and implications of moving to an online platform.

#### Theme 1: Expectations

RO-CBL surpassed many students’ initial expectations. Almost half (32/68, 47%) of the participants reported that RO-CBL was better than anticipated, stating that it was much smoother, easier to use, more enjoyable, and more practical than expected. Two participants stated they would be happy to use RO-CBL again, and 59% (40/68) disagreed with the statement “I would not like RO-CBL to be used in the future.”

I was a little hesitant to begin with however I enjoyed the experience and would use it again.

Thought it was going to be really bad, turns out was quite practical and have since used it for group assignments.

A total of 9 (13%) participants stated that RO-CBL met their expectations. One participant suggested this was due to adequate training prior to the learning activity; another had prior experience with video calls.

I thought it was pretty much what I expected from the lectures . . . I guess I've done . . . Skype calls and things like that before, so I knew how it was going to work and it . . . worked that way.

In contrast to this, 9% (6/68) of participants reported that RO-CBL did not meet expectations, stating that it did not run as smoothly as anticipated. One student suggested this might be due to poor Internet connectivity.

I just expected us to just do . . . a normal CBL case, read it out and have a bit more interaction. But it just turned out that we couldn’t do that, because . . . maybe it was just our Internet connections and everything like that, but it just didn't flow.

#### Theme 2: Benefits of RO-CBL

One significant benefit of RO-CBL is a flexibility that allowed participants to complete the CBL without having to be on campus. This had a significant impact on both time and costs associated with travel, including gas and road tolls.

I saved East Link [toll road], which up and back, that's $10 a day for me; and I saved petrol, which is probably $7, $7.50 a day for up and back as well. So that's $20, almost. I wouldn't mind that in my pocket.

This reduction in travel time gave participants an opportunity to complete other activities, including study, work and job seeking, exercise, and additional sleep.

Obviously [RO-CBL] is going to affect how much I spent on petrol and things like that as well; that I got to sleep in more, which was really good, and I got to spend that time that I didn’t spend travelling doing things like study and stuff like that, which was really good.

Three participants in the focus group and 4 in the open-ended questions noted that RO-CBL was more efficient compared to traditional face-to-face CBL. Participants proposed this might be due to decreased set-up time, a reduction in additional conversation, or not having to wait for someone to write notes on a whiteboard. These participants did not believe that the increased efficiency led to a decreased attention to detail during discussions.

The CBL could be done in a shorter time frame as you needed to get straight to the point and it was too difficult to have big chats or go off topic.

I think we were able to look at everything in detail. But just because no one was writing up, it just made it a bit faster.

#### Theme 3: Communication

Communication issues were noted during the RO-CBL by 9% (8/68) of participants in the open-ended questions. This was primarily due to Internet and microphone dropouts as well as microphone feedback between students. One participant suggested that this affected the quality of the CBL, and another suggested it affected the CBL’s efficiency.

Often . . . students . . . dropped out and then had to go back in, and we couldn't hear them, and the microphone wouldn't turn on properly. That really affected the quality, sometimes, of the CBL . . .

Some participants had difficulty with communication etiquette in the online environment. Participants recognized that video chat did not allow them to interpret body language, which meant that some hesitated when contributing to the discussion. It also meant that participants found themselves talking over one another because they could not predict when another group member was about to talk.

You don't want to start speaking at the same time, and then you're cutting other people off, and it's harder to hear both. So I just thought that . . . etiquette thing was something, if it was worked on a bit more, then it'd flow better.

The same number of participants stated that communication was easier than anticipated with RO-CBL.

I did not think initially that discussion would be possible with Internet dropouts however this was not the case.

There wasn’t an issue participating and taking turns. With further use it could improve as the novelty wears off and concentration increases.

#### Theme 4: Implications of Moving to an Online Platform

One implication of moving to an online platform is the possibility of difficulties associated with technology. Participants reported Internet disconnections that would result in their removal from the online workspace. Poor Internet connection also contributed to video and audio lag. Three participants recognized the need for more technical support. Other participants had no issues with Internet connectivity or other technical issues.

Whilst I liked doing CBL at home . . . people would log in and out due to technical difficulties, which made it hard.

The RO-CBL worked a lot better than I thought it would as a group we didn’t encounter any tech difficulties, which I thought we might.

Other issues associated with online learning identified by the participants included motivation and accountability. Three participants stated that they believed they learn better with face-to-face interactions compared to online due to increased motivation and decreased distractions. Two other participants suggested that online learning had less accountability resulting in fewer students contributing to discussions.

I think I'd learn better with a person, though. But everyone's different. Like if . . . yes, I don't think I'd learn as effectively just being on the computer the whole time.

There's also less accountability. It feels like you can tune out a bit more easily . . . because when you're all there in person sitting around a table you're quite accountable.

Additional cost is another complication of moving to an online platform. In order to participate in RO-CBL, students required Internet access and the necessary hardware, including a computer, microphone, and webcam.

I guess if everyone had a sort of a stable Internet connection, that . . . and like, really good webcam and really good microphones, that might be helpful. But I don't know how you'd do that without having a cost to the students . . .

Training is also necessary to operate in an online environment. Some participants felt that greater practice would resolve technical issues encountered during the RO-CBL. Others who had no technical issues stated that they were adequately prepared for the RO-CBL.

I thought Monash prepared us well for the ROCBL experience.

## Discussion

### Principal Findings

RO-CBL was generally well received by participants, with 59% (40/68) disagreeing with the statement “I would not like RO-CBL to be used in the future,” and 78% (53/68) of participants believing they could meet the CBL’s learning objectives via RO-CBL. This level of satisfaction contrasts with results from a pilot study by our research team, which found that 84% (16/19) of participants did not enjoy the Web-based activity and 73% (13/18) would not like to use the Web-based CBL in the future. However, results from this pilot study supported the notion that Web-conferencing CBL may provide students with a learning experience comparable to face-to-face CBL. This increase in student satisfaction could be due to the refined and improved implementation process of RO-CBL. Many participants found that RO-CBL exceeded expectations, suggesting that this might be due to the adequate training provided, while others reported that the learning activity only met expectations.

Those participants who found that RO-CBL did not meet expectations felt this was due to technical issues faced during the learning activity. Given that students completed the RO-CBL off campus, it is not surprising to find that some students experienced connection issues. This finding is consistent with other reports [8,9,12]. Participants noted that connection issues including microphone lag time and dropouts may have been detrimental to the flow of RO-CBL. Technical issues and difficulties associated with communication are common in Web-based learning [2,9,12]. Valaitis et al [12] recognized that the lag time and loss of conversational practices such as turn-taking and reference to previous statements creates challenges in online discussion. These issues were reported by a subset of students.

Participants recognized the flexiblity provided by RO-CBL. This is a common finding with Web-based learning [2,5,7,12]. Also important are the potential savings in financial costs and time for the student, which have not been previously quantified. These savings in time could also account for the perceived increase in efficiency students found with RO-CBL. Our preliminary data [18] indicate that user costs associated with RO-CBL are lower than costs for campus-based face-to-face CBLs ($6541 per student per semester compared to $7907). Cost from an institutional perspective has not been formally evaluated; however, given the reduction in space requirements, we anticipate RO-CBL will be found to be cost-effective. Cost is an important consideration when moving to an online platform, and further research is warranted.

Participants report benefits and obstacles with regard to RO-CBL. Although few participants reported technical issues relating to connectivity and appropriate hardware, this remains an ongoing issue. The most likely future is one where Internet access and connectivity improve and issues are resolved. Enabling learners through access to remote technical support may reduce connectivity issues, but these may also be resolved with anticipated advances in communication software.

Training appears to help learners operate in interactive, online environments and should be designed with consideration of learner needs [20]. Greenhalgh [5] highlighted that the amount of initial training for students to be comfortable using Web-based tools is often underestimated; our work supports the perceived benefits associated with opportunities to familiarize and troubleshoot with interactive environments. Participants felt that practice would resolve any technical issues. Learner needs might be assessed so training can target those who appear to be intimidated by the notion of Web-based learning [21]. Students need to adapt to communicating in these online environments; Valaitis et al [12] suggest there is a period of adaptation before students engaged in meaningful online PBL discussions. This might be facilitated by well-designed training.

### Limitations

Students had two years of previous face-to-face CBL experience, so training was targeted at transferring those skills to an online environment. Care was taken within this study to minimize the impact of specific Web conferencing software on the results. To achieve this, only the features common across all Web conferencing platforms were used. Despite these efforts, it remains possible that the results may be different with an alternative program. Learning outcomes were not assessed directly in this study.

### Conclusion

Web conferencing may be a suitable medium for student participation in CBL. Participants were satisfied with the learning activity and felt they could meet the CBL’s learning objectives, which may be due to the training provided. While there are benefits to RO-CBL, obstacles remain. Ensuring students have remote technical support and adequate Internet connection are challenges that need to be addressed to successfully implement any remote learning activity. Targeted training is necessary to ensure students are comfortable operating and communicating in the online environment. It is hypothesized that these issues will be overcome once the students adapt to the online environment, but this needs further investigation. Further study should also evaluate Web conferencing CBL across an entire semester in regard to student satisfaction, perceived depth of learning, and learning outcomes.

## Abbreviations

CAL: computer-assisted learning
CBL: case-based learning
PBL: problem-based learning
RO-CBL: remote-online CBL
WBL: Web-based learning
